# Singing abilities in children with Specific Language Impairment (SLI)

**DOI:** 10.3389/fpsyg.2015.00420

**Published:** 2015-04-13

**Authors:** Sylvain Clément, Clément Planchou, Renée Béland, Jacques Motte, Séverine Samson

**Affiliations:** ^1^Neuropsychology: Auditory, Cognition, Action Team, Laboratoire PSITEC, UFR de Psychologie, Université de LilleVilleneuve d'Ascq, France; ^2^Neurologie Pédiatrique, Pôle Femme-Mère-Enfant, American Memorial HospitalReims, France; ^3^École d'Orthophonie et d'Audiologie, Université de MontréalMontréal, QC, Canada; ^4^Unité d'Épilepsie, Groupe Hospitalier Pitié-SalpêtrièreParis, France

**Keywords:** Specific Language Impairment, musical perception, musical production, singing, pitch-matching

## Abstract

Specific Language Impairment (SLI) is a heritable neurodevelopmental disorder diagnosed when a child has difficulties learning to produce and/or understand speech for no apparent reason (Bishop et al., 2012). The verbal difficulties of children with SLI have been largely documented, and a growing number of studies suggest that these children may also have difficulties in processing non-verbal complex auditory stimuli (Corriveau et al., 2007; Brandt et al., 2012). In a recent study, we reported that a large proportion of children with SLI present deficits in music perception (Planchou et al., under revision). Little is known, however, about the singing abilities of children with SLI. In order to investigate whether or not the impairments in expressive language extend to the musical domain, we assessed singing abilities in eight children with SLI and 15 children with Typical Language Development (TLD) matched for age and non-verbal intelligence. To this aim, we designed a ludic activity consisting of two singing tasks: a pitch-matching and a melodic reproduction task. In the pitch-matching task, the children were requested to sing single notes. In the melodic reproduction task, children were asked to sing short melodies that were either familiar (FAM-SONG and FAM-TUNE conditions) or unfamiliar (UNFAM-TUNE condition). The analysis showed that children with SLI were impaired in the pitch-matching task, with a mean pitch error of 250 cents (mean pitch error for children with TLD: 154 cents). In the melodic reproduction task, we asked 30 healthy adults to rate the quality of the sung productions of the children on a continuous rating scale. The results revealed that singing of children with SLI received lower mean ratings than the children with TLD. Our findings thus indicate that children with SLI showed impairments in musical production and are discussed in light of a general auditory-motor dysfunction in children with SLI.

## Introduction

Children with Specific Language Impairment (SLI) are characterized by persistent expressive and receptive language difficulties with normal intelligence, normal hearing, and the absence of apparent neurological damages. This developmental disorder affects 2–7% of the population (Tomblin et al., 1997; Law et al., 1998). The language problem often persists through adolescence and may have long-term negative consequences at emotional and behavioral levels (Conti-Ramsden and Botting, 2008). Although SLI is heritable, it is the result of a complex interaction between genetic and environmental risk factors (Bishop, 2006).

According to the DSM-IV (American Psychiatric Association, 2000), the diagnosis requires the language abilities to be below the expected level according to the age and the non-verbal performance of children with Typical Language Development (TLD) using conventional cut-offs between 1 and 1.25 standard deviations below the mean. Hearing loss, autistic spectrum disorders, intellectual disabilities are all exclusion criteria for a diagnosis of SLI. The language difficulties typically affect grammatical, syntactical, and phonological processing to a level that severely impedes communication. However, children diagnosed with SLI present heterogeneous profiles in their language deficits (Bishop, 2001). They may also present auditory processing deficits (Corriveau et al., 2007; Bishop et al., 2010) as well as more general cognitive impairments in working memory (Gathercole and Baddeley, 1990) or procedural memory (Ullman and Pierpont, 2005). These heterogeneous profiles of deficits raise the question as to whether or not SLI is a single syndrome.

Three theoretical accounts for deficits in children with SLI can be distinguished. First, according to the linguistic account, a subgroup of children with the “Grammatical-SLI” exhibit a domain specific impairment that affects the processing of complex linguistic structures at syntactical, morphological, and phonological levels (Marshall and van der Lely, 2007). The cognitive account proposes that SLI mainly results from deficits in auditory working memory (Gathercole and Baddeley, 1990) or in procedural memory (Ullman and Pierpont, 2005). In the auditory account, difficulties of the children with SLI in the non-word repetition task would result from impaired short-term auditory memory (Gathercole and Baddeley, 1990). This impairment would extend to the whole working memory system (for a review see Montgomery, 2003). The procedural memory deficit that explains language impairments has led Ullman and Pierpont (2005) to hypothesize that the children with SLI would exhibit cerebral abnormalities in brain regions involved in procedural memory, mainly the frontal lobe structures and the basal ganglia. This hypothesis is supported by several brain imaging studies reporting structural singularities in children with SLI in both inferior frontal regions including the Broca's area (Gauger et al., 1997; Clark and Plante, 1998; Vargha-Khadem et al., 1998) and the basal ganglia (Jernigan et al., 1991; Vargha-Khadem et al., 1998). In order to investigate the presence of a procedural memory deficit in these children, Lum et al. (2012) compared 51 children with SLI and 51 children with TLD in working, declarative and procedural memory tasks. Results confirmed that both auditory working memory and procedural memory were severely and independently impaired in children with SLI, whereas visuo-spatial short-term memory and both verbal and visuo-spatial declarative long-term memory were spared. The third account, the auditory account, postulates that a low-level auditory processing disorder is responsible for SLI (Rosen, 2003; Moore, 2006; Dawes and Bishop, 2009). As in developmental dyslexia, children diagnosed with SLI have been found to show difficulties in rapid auditory temporal processing (Benasich and Tallal, 2002), rise-time contrast discrimination (Corriveau et al., 2007), and sound duration discrimination (Friedrich et al., 2004; Corriveau et al., 2007). Furthermore, frequency processing could also be impaired in SLI (Korpilahti, 1995; Hill et al., 2005; Mengler et al., 2005; Nickisch and Massinger, 2009; Bishop et al., 2010). One argument against the low-level auditory processing hypothesis is that a large proportion of individuals with SLI perform within the normal range in auditory processing tasks (Bishop et al., 2005; McArthur and Bishop, 2005), thereby suggesting that SLI can be present in absence of auditory processing deficits (Bishop et al., 1999). Thus, Bishop et al. (2012) recently suggested that the auditory deficits are the consequence rather than the cause of SLI. All these findings question the existence of other auditory processing deficits in children with SLI. Such difficulties would likely extend to the musical domain by impacting not only perception but music production as well.

An increasing number of studies have suggested that a close relationship exists between language and music functions (Besson and Schön, 2003; Koelsch and Friederici, 2003; Patel, 2008). Functional brain imaging studies have shown that the cerebral regions underlying speech and music processing overlap in adults (i.e., Tillmann et al., 2006). The possible relation between verbal and musical abilities is also supported by behavioral data. Jones et al. (2009) screened a large population of participants (864 people aged from 15 to 60 years) with no history of language deficit. They tested all participants using the “Distorted Tunes Test” (Kalmus and Fry, 1980). In this task, the participants were asked to detect a pitch change in popular tunes and to report whether the melody was familiar or not. Results revealed that 35 participants (4% of the total population sample) were significantly impaired in this task. Interestingly, the authors showed that these musical difficulties were associated with deficits in phonological awareness, which thus indicates there to be an intricate relationship between music and language processing. This finding is consistent with Anvari et al.'s (2002) hypothesis that music perception employs auditory mechanisms that are partially overlap with those involved in phonological awareness. In a recent review, Brandt et al. (2012) emphasized the parallel development of language and musical abilities based on the result of a study reporting similar electrophysiological markers of syntax processing in language and music (Jentschke et al., 2008). Taken altogether, these results suggest that language and musical abilities can be impaired at the same period during child development. We therefore predict that language disorders in children with SLI might co-occur with musical difficulties.

Another group of studies support the hypothesis of independent processing of language and music. First, Mottron et al. (1999) reported a dissociation between language and music abilities in an 18-year old autistic woman with absolute pitch. This person showed severe deficits in language perception and production, despite a normal pitch perception performance compared to healthy controls with absolute pitch. In line with this observation, Alcock et al. (2000) revealed that nine members of the KE family with inherited language disorder showed spared performance in pitch and melodic discrimination tasks (but not for rhythm discrimination). The reverse dissociation has recently been reported in children with congenital amusia (Lebrun et al., 2012; Mignault Goulet et al., 2012), a syndrome defined as a disorder of music processing that is not explained by intellectual or sensory deficits (Peretz, 2001; Ayotte et al., 2002). The musical deficits are generally evaluated using the Montreal Battery of Evaluation of Musical Abilities (MBEMA, Peretz et al., 2013). Lebrun et al. (2012) described a 10-year old girl who was impaired in all musical subtests of MBEMA (melody, rhythm and memory subtests) whereas her verbal abilities were unimpaired. In a subsequent study of eight children with congenital amusia (10–13 years old), Mignault Goulet et al. (2012) found that they all had deficits in melodic discrimination and, to a lesser degree, in rhythm discrimination despite the fact that they did not present signs of language disorders. All together, these results support a possible double dissociation between linguistic and musical abilities.

In another study (Planchou et al., under revision), we found that a large proportion of children with SLI showed impairments in the MBEMA (Peretz et al., 2013), which indicates that language impairments are frequently associated with difficulties in music processing. Given that, in children with SLI, language is impaired in both perception and production tasks, we predicted that musical deficits in children with SLI should impact not only perception but also musical production, such as singing. Indeed, impairments in music perception should logically affect singing ability since this activity may require an accurate perception of its own voice in order to precisely adjust its pitch. Although this link between musical perception and production has been often reported (see for example Dalla Bella et al., 2007), dissociations have also been documented. First, poor singing can co-occur with unimpaired musical perception (Berkowska and Dalla Bella, 2009). More surprisingly, the reverse dissociation (spared singing despite impaired music perception) is also supported by several studies. Loui et al. (2008) showed that people with amusia may be able to reproduce pitch directions by singing despite being unable to detect the same pitch directions. Consistent findings have been reported by Dalla Bella et al. (2009) in five cases of amusia, who displayed spared abilities in reproducing pitch directions. Moreover, two of them were able to sing with lyrics as proficiently as control participants. These different results raise the question of whether children with SLI also exhibit impaired singing abilities.

To our knowledge, no study has yet investigated the singing abilities in children with SLI. In a preliminary unpublished experiment, we found very severe singing deficits in five children with SLI who were completely unable to produce a very familiar tune (“Brother John”). The recorded data were impossible to analyze because the children could not sing at all, which suggests that children with SLI may present difficulties with not only spoken language but also with singing abilities. Given that the singing deficits might have been exacerbated by the potentially stressful experimental conditions, we designed a new experiment involving a ludic activity for the present study. The singing ability of each child was individually tested while playing a game (“Game of the goose”) with the experimenter. The task consisted of singing either single notes (pitch-matching task) or melodies (melodic reproduction task) on the syllable (*/la/*) immediately after presentation. In the Melodic Reproduction task, three types of melodies were used to control for the effects of familiarity with musical excerpts and the influence of lyrics in singing. In the FAM-SONG condition, two familiar songs learned with lyrics were used (“Brother John” and “Au clair de la lune”). In the FAM-TUNE condition, two familiar melodies with no associated lyrics were taken from musical pieces used in TV cartoons or movies (“Pink Panther theme” and “Mission: impossible”). Finally, two new melodies were composed for the UNFAM-TUNE condition. We also assessed musical perception in all children using the MBEMA (Peretz et al., 2013) in order to examine the possible relation between production and perception abilities in the musical domain in children with SLI. According to our hypothesis, children with SLI were expected to present difficulties in both singing isolated notes as well as singing short melodies compared to children with TLD. In both groups, singing familiar melodies (FAM-SONG and FAM-TUNE conditions) were expected to be easier than singing unfamiliar melodies (UNFAM-TUNE). However, considering the language deficits in children with SLI, singing songs previously learned with lyrics (FAM-SONG condition) was expected to increase their difficulties.

## Materials and methods

### Participants

Eight children with SLI (two girls and six boys) were recruited from schools with special education programs for language disorders, in Reims and Charleville Mézières (France), and 15 (six girls/nine boys) children with TLD also participated in this study. No children in the TLD group had a history of verbal or musical disorder, and none received speech therapy. In both groups, no children had physiological, psychiatric, or neurological problems, none received musical training, and their native language was French.

The two groups were matched for age, sex, and non-verbal intelligence (Table 1). To assess non-verbal intelligence in the group of children with SLI, we used the scores reported in the latest clinical report, which was either the score in the Performance subtests of the Wechsler Intelligence Scale for Children (WISC-III; Wechsler, 1991), the score in the Perceptual Reasoning Index (WISC-IV, Wechsler, 2003), or the score in the Colored Progressive Matrices test (Raven et al., 2003). In the group of children with TLD, non-verbal intelligence was assessed with the Colored Progressive Matrices test. As reported in Table 1, non-verbal intelligence, expressed in percentile, was within the average range (>ninth percentile) for participants in both groups. The SLI group and TLD group did not differ in age [*t*_(21)_ = 0.1; *p* = NS] or non-verbal intelligence scores [*t*_(21)_ = 0.9; *p* = NS]. All the children were also assessed with the MBEMA (Peretz et al., 2013). The results revealed that the SLI group scored lower than the TLD group (see Table 1 for details). Moreover, all the children with TLD had normal scores in the MBEMA whereas five children with SLI were impaired in this test, according to their global score (Table 2).

**Table 1:** **Characteristics of the children from the SLI and TLD groups**.

		**SLI (*N* = 8)**			**TLD (*N* = 15)**			**Comparison**	
		***M***	***ET***	**Range**	***M***	***ET***	**Range**	***t*_(21)_**	***p***
**Age**		11.0	1.6	8.7–12.9	11.1	1.5	8.2–12.9	0.1	0.9
**Non-verbal intelligence (percentiles)**		35.9	22.2	9.0–75.0	47.3	30.1	10.0–95.0	0.9	0.4
**MBEMA**	Global score (percent)	70.8	12.5	55.0–90.0	86.7	7.6	73.3–96.7	3.8	<0.01
	Melodic subtest (/20)	12.2	3.0	10.0–17.0	16.5	2.6	12.0–20.0	3.6	<0.01
	Rhythmic subtest (/20)	15.0	3.5	9.0–19.0	18.6	1.1	16.0–20.0	3.7	<0.01
	Memory subtest (/20)	15.2	1.5	14.0–18.0	16.9	2.2	13.0–20.0	1.8	0.08

**Table 2:**
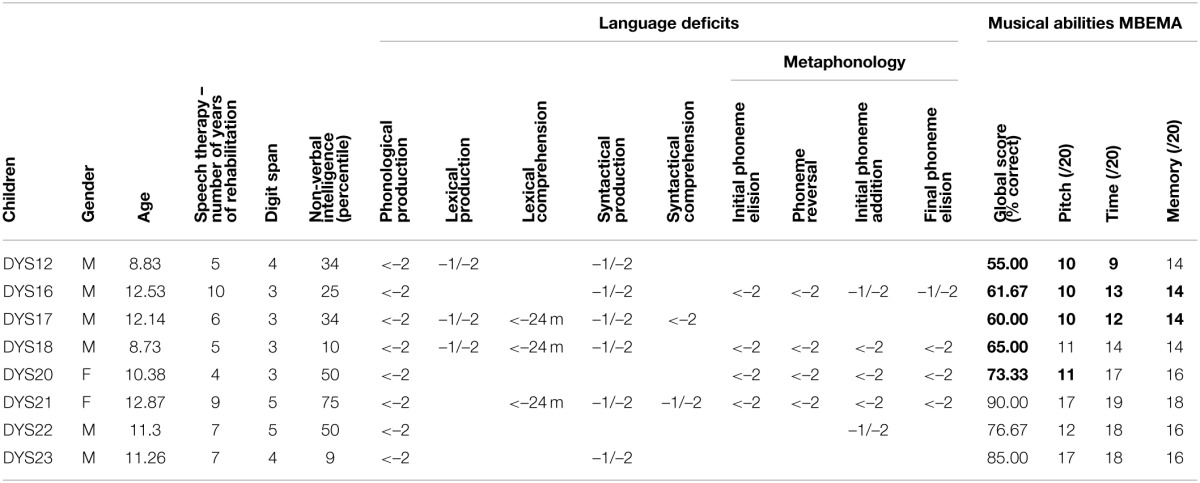
**Music perception and language data for children in SLI group**.

*Language: scores below 2 SD (<–2) or between 1 and 2 SD (–1/–2) from the normal control scores, scores 24 months (<–24 m) below the level expected, empty cells correspond to normal scores. Music: impaired scores according to Peretz et al. (2013) are displayed in bold*.

Children with SLI had been enrolled in speech therapy for 4–10 years. They suffered from deficits affecting both expressive and receptive language at different levels of severity. They scored more than one standard deviation (or 18 months) below the level expected for their age on at least two of the six spoken language tests, as displayed in Table 2.

Finally, all participants were administered a hearing test, whereby sounds were presented in both the left and right ear at a range of frequencies (125, 250, 500, 1000, 2000, 4000 Hz). All children were sensitive to sounds within the 20 dB HL range, thus confirming the absence of hearing impairment in both groups.

### Procedure

Each participant played a computerized version of the “Game of the goose” against the experimenter. Each player's avatar was moved along the track according to throws of a virtual dice (computer simulated). The winner was the first player to reach the end of the game board. Each time a participant landed on a blue space, he had to perform a singing task: either to sing an isolated musical note (pitch-matching task) or to sing a melodic sequence (see below for the complete description of these tasks). The simulated dice was “unfair” in order to allow the children to win the game, to get the same number of sung productions from each child, and an equal number of sung productions from the child and from the experimenter. This precaution was taken in order to maintain a motivating competition between the child and the experimenter. The order of all the stimuli to be sung during the game (isolated notes and melodies) was randomized and identical for all the children.

#### Pitch-matching

In each “pitch-matching” trial, a musical note played with a piano sound was presented twice to the participant through headphones. After each presentation, the participant had to sing back the note on the */la/* syllable. Over the entire game, the participants had to sing all the chromatic degrees starting from C_4_ (*f*_0_ = 261.23 Hz) to B_4_ (*f*_0_ = 493.88 Hz).

#### Melodic reproduction

In the “melodic reproduction” trials, the participant listened to a melody played twice with a piano sound. After each presentation, the players (the child or the experimenter) had to sing it back on */la/* syllables. Over the entire game, the participants had to sing a total of six melodies: two familiar ones that are generally associated with lyrics (FAM-SONG condition: “Brother John,” “au clair de la lune”), two familiar ones that are normally not associated with lyrics (FAM-TUNE condition: “Pink Panther,” “Mission: Impossible”) and two unfamiliar ones composed for the study (UNFAM-TUNE condition: “UnknownA,” “UnknownB”). The familiar melodies consisted of the first musical phrase of the main theme, lasting 4–5 bars. The unfamiliar tunes were of the same length and had the same rhythmic and melodic complexity as well as the same pitch range as the familiar ones. The familiar tunes and songs were selected after a survey in a school with children aged 6–10 years. In the FAM-SONG and FAM-TUNE conditions (familiar melodies), the title of the tune was announced to the player before the example was played and, after the experiment, we verified that all children were familiar with the tune.

### Stimuli and material

**Figure 1:**
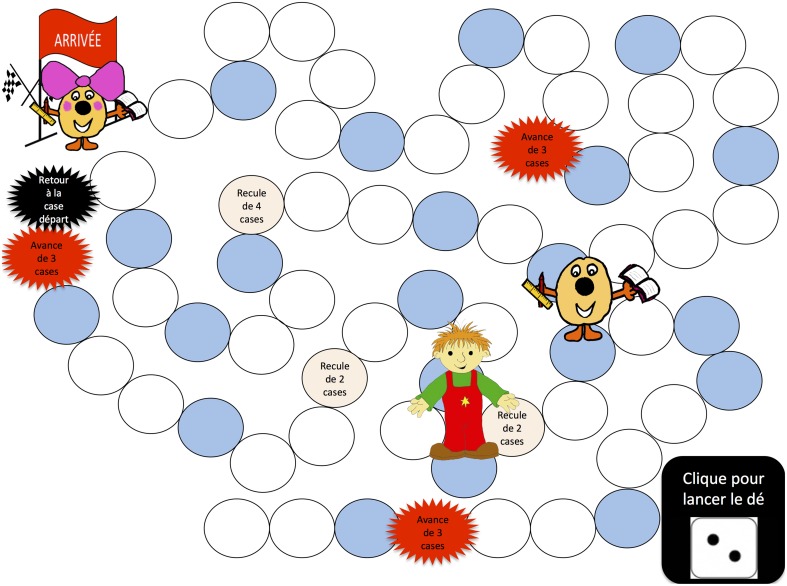
**Screen capture of the “game of the goose” used for assessing singing abilities of children**.

The game board (Figure 1) was depicted on a laptop computer screen and the dice throwing was also computer simulated. The board consisted of a track of consecutive spaces. Eighteen blue spaces were scattered around the board.

The sample notes for the pitch-matching task were produced using the “Steinway Grand Piano” virtual instrument of Apple Logic Pro 9 software. All the chromatic degrees of the equal-tempered scale had been produced starting from C_4_ (*f*_0_ = 261.23 Hz) up to B_4_ (*f*_0_ = 493.88 Hz) with a mean length of 1.4 s. The melodies for the Melodic Reproduction task were produced using the same configuration. All notes of the melodies had been entered by hand with a fixed MIDI velocity of 80 and a tempo of 120 bpm resulting in a mean melody length of 7.02 s (*sd* = 1.9 s).

All the gaming activity was recorded with a Zoom H2 digital audio recorder (uncompressed WAV file type, 16 bits/44.1 kHz) placed just in front of the children.

### Scoring of productions

All the sung productions (both single notes and melodies) were extracted from the continuous recording for evaluation.

#### Pitch-matching accuracy

The pitch of each note was computed using and the ProsodyPro script (Xu, 2013) in the Praat software (Boersma, 2001). The pitch was then compared to the target pitch to compute absolute errors (in cents) to prevent sharp and flat errors from canceling each other out.

#### Melodic reproduction evaluation methodology

On an initial listening, the singing production of children with SLI sounded severely impaired, to the point that we could not recognize the target tunes. An acoustic analysis, as the one we used in the pitch-matching task would therefore have been useless since it would not have been possible to align the target tune with the child's production. We thus opted for a subjective evaluation of the melodic reproductions, a method that has proved to be consistent with acoustic analysis and previously validated (Larrouy-Maestri et al., 2013). All the reproduction of melody examples produced by the children were sorted by tune and presented to 30 healthy judges (age: mean = 26.8 years, *sd* = 5.3; less than 2 years of formal musical education) for a subjective evaluation on a continuous scale. Each judge had to rate the 46 productions (23 children; two trials) of two out of six tunes, and so each production was thus rated by 10 judges. Judges were not aware of the presence of recordings from children with SLI, and were thus blinded from group affiliation. The judges heard the example that was given to the children before each reproduction. They were then instructed to rate each production on a continuous scale running from 1 to 10, taking into account the accuracy of both pitch and rhythm. They also were explicitly asked to ignore any global transposition of the melodies. Moreover, the mean inter-rater reliability between judges, as measured by Spearman correlation coefficient, was good (ρ = 0.79). All the ratings were averaged in order to calculate a mean rating for each child in each condition.

## Results

### Pitch matching

**Figure 2:**
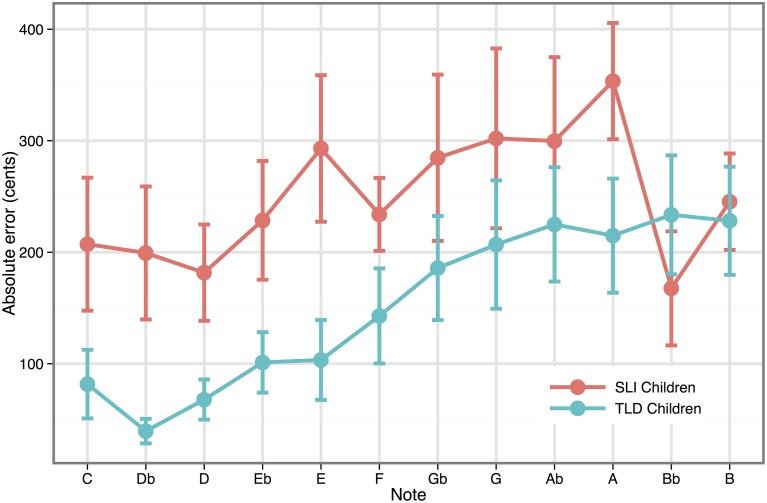
**Mean absolute errors (in cents) in the pitch-matching task as a function of target note**.

A Two-Way ANOVA with Target note (12 notes from C_4_ to B_4_) as a repeated measure was carried out on the absolute errors in cents of the reproduction of single notes, for the group of children with SLI and the group with TLD. Results were computed with Greenhouse–Geisser corrections when the assumptions of sphericity were violated. As displayed in Figure 2, the error values in cents were greater in the group of children with SLI than in the group of children with TLD. The finding was borne out by statistical analysis that revealed an effect of Group [*F*_(1, 21)_ = 4.41, *p* < 0.05], the SLI group producing larger pitch-matching errors than the TLD group (mean errors: SLI = 249.74 cents, TLD = 152.58 cents). A significant effect of target note was also found [*F*_(11, 231)_ = 4.52, *p* < 0.005], whereby pitch accuracy decreased as target pitch increased. However, the Group by Target note interaction was not significant [*F*_(11, 231)_ = 1.37, *p* = 0.25].

### Melodic reproduction

**Figure 3:**
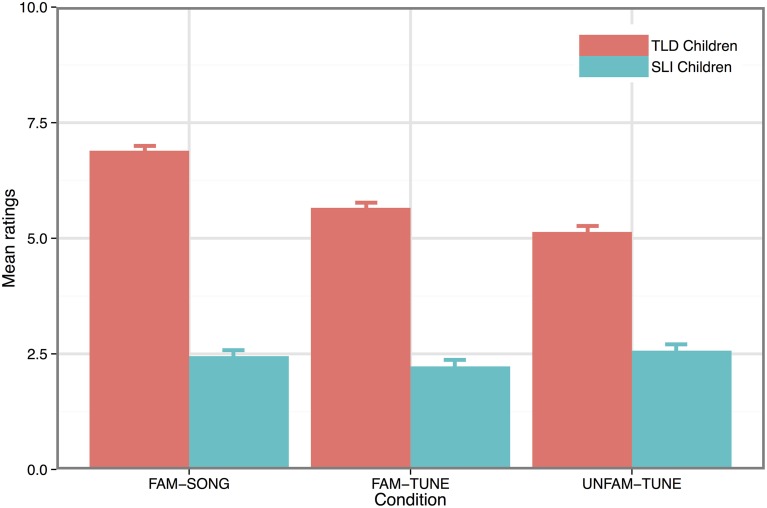
**Mean ratings in the melodic reproduction task as a function of experimental conditions**.

Figure 3 displays the mean ratings in the melodic reproduction task as a function of Group and Condition. After checking the assumptions, an ANOVA was run with Group (SLI vs. TLD) and Condition (FAM-SONG, FAM-TUNE, UNFAM-TUNE) as factors. This revealed a significant effect of Group [*F*_(1, 21)_ = 31.03, *p* < 0.001] and Condition [*F*_(1, 42)_ = 8.0, *p* = 0.002] as well as a significant Group by Condition interaction [*F*_(1, 42)_ = 4.24, *p* = 0.02]. *Post-hoc* analysis with Holm-Bonferroni correction revealed no differences between the conditions in the SLI group whereas, in the TLD group, the ratings were significantly higher in the SONG than in the UNFAM-TUNE condition (the TUNE condition lying between these two).

### Correlations between musical production, musical perception and language abilities

In order to relate accuracy of pitch-matching to abilities in music perception measured in the MBEMA, a mean pitch error was computed by averaging the absolute pitch errors for the three lowest notes (C, Db, and D) for which the performance was optimal. We found a strong inverse correlation (Spearman's method) between the mean pitch error and the global MBEMA score (ρ = −0.73, *p* < 0.001) as well as with the melodic (ρ = −0.79, *p* < 0.001) and the rhythmic (ρ = −0.73, *p* < 0.001) subtests for all participants. The correlation between mean pitch error and the memory subtest of the MBEMA scale was not significant (ρ = −0.37, *p* = 0.07). When looking for these same correlations within each group, we found that the mean pitch error of children with TLD only correlated significantly with the melodic (ρ = −0.67, *p* = 0.006) and rhythmic (ρ = −0.54, *p* = 0.04) subtests of the MBEMA scale. No correlations were found between the mean pitch error of the children with SLI and the global MBEMA score or the subtests scores.

**Figure 4:**
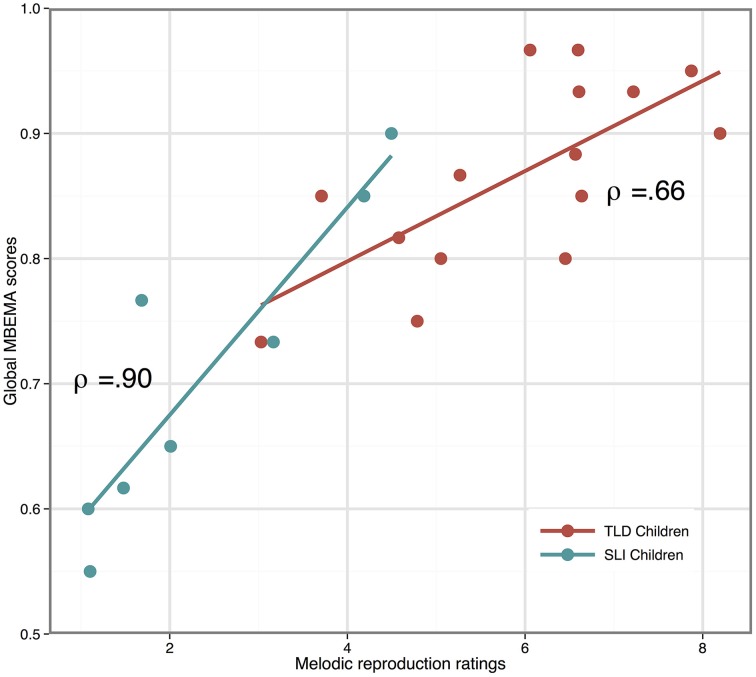
**Scatter plot illustrating the correlation between musical production (melodic reproduction task) and musical perception (MBEMA scores)**. Regression lines are fitted for each group and the spearman's ρ are indicated.

The Spearman's correlation test revealed a strong positive correlation between the mean ratings in the melodic reproduction task and the global MBEMA score (ρ = 0.82, *p* < 0.001) as well as for the melodic (ρ = 0.87, *p* < 0.001), the rhythmic (ρ = 0.78, *p* < 0.001), and the memory (ρ = 0.50, *p* = 0.01) subtests of the MBEMA. All of these correlations reached the significance level within each group with the exception that the mean ratings of children with TLD did not correlate significantly with their performance in the Memory subtest of the MBEMA. Figure 4 depicts the correlation between mean ratings and MBEMA Global score for each group.

As predicted, we found a strong negative global correlation between the mean ratings in the melodic reproduction task and the mean value in cents in the pitch-matching task (ρ = −0.76, *p* < 0.001). At the group level, this correlation only reaches significance for the children with TLD (ρ = −0.73, *p* = 0.003).

For children with SLI, we also examined correlations between musical production and language skills. Two composite scores were computed for their receptive and productive language performance. The “receptive” score was computed by averaging the proportions of correct responses for lexical and syntactic comprehension tasks. The “productive” score was computed by averaging the proportions of correct responses for the phonological, lexical, and syntactic production tasks. None of these scores correlated with the performance in the pitch-matching task or the melodic reproduction task. We also failed to find a correlation between the metaphonological performance of children with SLI and performance of the two singing tasks.

### Individual analysis

Individual analyses were run in order to estimate the proportion of children with SLI that were impaired in each task and to question the presence of a deficit in both perception and production. Because of the small number of children in the control group (*n* = 15), we could not use the classical *z*-score method for individual analyses. Instead, we used the procedure described by Crawford and Garthwaite (2007) that allows each individual in the SLI group to be compared with the children in the TLD group. The results revealed that six out of eight children with SLI were significantly impaired on the melodic reproduction task. The two children with SLI that were not impaired were also those who had the highest global scores in the MBEMA and that were not significantly impaired in musical perception according to the MBEMAn (subjects DYS21 and DYS23). Results of the pitch-matching task were also analyzed using the procedure by Crawford and Garthwaite (2007), which revealed that none of the children with SLI taken individually were significantly impaired compared to the group of children with TLD.

## Discussion

The present study aimed to investigate whether expressive language impairments in children with SLI extend to musical production, which was here evaluated by two singing tasks. The participants played a “game of the goose” with the experimenter, which provided a motivating, pleasant testing environment. This allowed us to record the sung productions of eight children with SLI, which may not have been possible in a classical experimental setup as suggested by our first trials (unpublished). Our results showed that children with SLI were less accurate than the children with TLD in reproducing isolated notes and short melodies. These group differences could not be attributed to differences in age or non-verbal intelligence because the two groups were matched on these variables. We also confirmed the presence of a musical perception deficit in children with SLI as measured by the MBEMA scale (Peretz et al., 2013). Among the eight children with SLI who participated in this study, six showed a deficit in singing melodies, which was (for five out of six of them) associated with a deficit in musical perception.

In the pitch-matching task, the absolute error was measured in cents. The analysis showed that the mean error value (in cents) was larger in the SLI than in the TLD group. This result seems to contradict with the results of Alcock et al. (2000), who report results from the language-impaired members of the KE family. Alcock et al. (2000) reported no difference in pitch-matching abilities between the KE family members and controls. The lack of differences between the two groups of participants in their study could be explained by the method they used to analyze the results; the performance of both groups in the pitch-matching task was expressed using the proportion of correct responses. According to their definition, a response is correct when the pitch-matching error is less than one half-tone (100 cents). Using the same method, the mean proportion of correct response in the present study would be equal to 0.64 (*sd* = 0.43) for the children with SLI. Therefore, the performance of children with SLI would not differ from the performance of the KE members (mean proportion of correct responses = 0.60). However, the TLD group in our study obtained a mean proportion of correct responses of 0.86 (*sd* = 0.32), which was higher than the proportion of correct responses obtained by the control participants in Alcock et al.'s study (mean value = 0.75), despite the participants in their control group were older (mean age = 18.3 years) than the children in our TLD group (mean age = 11.1 years). Considering that we carefully excluded children with musical education, these discrepancies remain difficult to explain. Interestingly, the results of children with SLI in our study appear to be more similar to those reported in congenital amusia by Hutchins et al. (2010) conducted with older participants (age range = 57–70 years). By using a pitch-matching task, these authors reported a mean error score of of people with congenital amusia (200 cents) that was lower than the mean error score of children with SLI in our study (249.8 cents). However, the control adults in Hutchins et al.'s study performed much better (mean pitch error < 25 cents) than the children with TLD in our study (mean pitch error = 153 cents), thus confirming an age effect in the development of singing abilities (Rutkowski, 1997; Welch, 2009).

Despite our efforts to reduce the level anxiety of children with SLI in singing tasks, their sung productions were rated lower than those of the children with TLD. While this result is in agreement with our hypothesis, it contradicts the results reported by Alcock et al. (2000) who showed that KE family members did not perform worse than the control participants when they were requested to sing melodies. A possible explanation is that our task required no verbal production, except the syllable */la/*. Considering that children with SLI have difficulties in expressive language, we hypothesized that lyrics would interfere with melodies. The children in our study were therefore instructed to sing all the melodies on the syllable */la/*. Note, however, that Dalla Bella et al. (2009) reported that for amusics, singing on the syllable */la/* seemed harder than singing with lyrics. According to this view, in our study, singing in children with SLI might have been hampered by the use of a single syllable rather than uttering the words. This interpretation seems rather unlikely since we showed, in a preliminary study, that singing with lyrics appeared to be more difficult for children with SLI than singing on the syllable */la/*. A second explanation for the discrepancies found between the results of our study and those of Alcock et al. is the difference between the tasks. In our study, we presented the whole melody before asking the children to reproduce it, whereas in Alcock et al.'s (2000) study, the title or the first melodic phrase was presented and the participants were then requested to sing or to continue by themselves. Their task was thus not a simple melodic reproduction task in that it involves long-term memory components that might have contributed to decrease the differences between the members of the KE family and the control participants. In our study, 30 healthy participants who were blind to experimental conditions rated the melodic reproductions. We are confident that our findings suggest that children with SLI presented difficulties in singing in addition to their difficulties in expressive language.

The individual analyses of the performance of children with SLI in the melodic reproduction task revealed that six out the eight children with SLI were impaired in this task compared to children with TLD. The two children with SLI who showed no impairments (DYS21 and DYS23) were two children who were not impaired in musical perception, as attested by their good performance in the MBEMA (Peretz et al., 2013). One of the children with SLI (DYS22) was significantly impaired in the melodic reproduction task but not in the MBEMA. Although no strong conclusions could be drawn on the basis of this single case, it suggests that more severe deficits in musical abilities in children with SLI are to be observed when a task relies on production rather than music perception abilities. Such a profile of responses has also been reported in the verbal domain, children with SLI having greater difficulties in expressive than in receptive language (Gérard, 1993). It has also been demonstrated in some amusic participants, who seem to be more severely impaired in a singing task than in musical perception tasks (Dalla Bella et al., 2009)–although the reverse dissociation was also found by the authors. This apparent dissociation revealed in participant DYS22 may also be explained by the difference in the nature and difficulty of the tasks. Further studies are required before drawing firm conclusions.

Three conditions were proposed in the melodic reproduction task. Our analysis revealed that children with TLD performed better in singing familiar songs than unfamiliar melodies (the familiar melody performance being in between the two). This suggests that children with TLD may take advantage of both the familiarity of the melodies and the lyrics associated with the melodies in the FAM-SONG condition. On the contrary, children with SLI did not benefit from the familiarity of the melodies or from the lyrics associated to them. They were not better in singing familiar than unfamiliar melodies, which suggest that activation of lyrics did not help them to reproduce the melody. However, considering the small number of children in the SLI group, and considering that their low performance could have lead to floor effects, we cannot decipher whether the activation of lyrics did indeed increase their deficits in the FAM-SONG condition of the melodic reproduction task.

Strong correlations were found between the global score in the MBEMA and the performance in both the pitch-matching and melodic reproduction tasks. In the pitch-matching task, the mean error in cents correlated with the scores in the melodic and rhythmic subtests but not with those in the memory subtest. The melodic and rhythmic subtests consisted, in each trial, of a same-different discrimination task between two short melodies. Although short-term memory may be involved, these subsets mainly evaluate perception whereas the memory subtest is a recognition task that tests whether the short melodies used in the previous subtest were incidentally encoded in long-term memory. In the melodic reproduction task, the mean rating values were strongly correlated with all the subtests of the MBEMA, including the memory subtest. The latter result makes sense considering the mandatory involvement of short-term memory processes in memorizing the melodies before reproduction. The fact that the deficits in music perception and production were strongly correlated in children with SLI suggests that these deficits resulted from an impairment affecting processes that are common to both music production and perception. Berkowska and Dalla Bella (2009) reviewed the plausible explanations of poor singing with reference to the functioning of the vocal sensorimotor loop. They argued that impaired perception hindered appropriate monitoring of the ongoing vocal performance and may lead to altered accuracy of singing. However, Berkowska and Dalla Bella (2009) also underlined that poor singing may be explained by alteration of non-perceptual processes such as memory and motor planning and execution. This raises the question as to whether the singing deficits of children with SLI can be solely explained by perception deficits or whether they are amplified by other dysfunctions in the vocal loop.

While the sung production in the pitch-matching task was evaluated with objective measures of pitch accuracy, this was not the case for the melodic reproduction task. At first listening, the deficits of the children with SLI were so obvious that it was impossible to carry out acoustic analysis and, as such, we used subjective ratings. While such an acoustical analysis could have provided fine measurement of pitch, rhythm and contour accuracy, significant parts of productions of children with SLI were not even recognizable. Until recently, the subjective evaluation of sung production has been the dominant approach when evaluating singing in neurological patients (Berkowska and Dalla Bella, 2009). Furthermore, Larrouy-Maestri et al. (2013) recently documented a strong correlation between objective and subjective measures of singing. To prevent rater bias, judges were not aware that production was also measured in children with SLI. Therefore, the lower ratings obtained by the children with SLI could not be explained by any bias in the subjective evaluation. The blinded judges were only instructed to rate the performances taking into account relative pitch accuracy (thus allowing for transposition) and rhythm. Despite such minimalistic instructions, the mean inter-raters reliability was good (Spearman's ρ= 0.79), which suggests that these subjective evaluations provide a reliable measure of singing performance of children.

According to our results, children with SLI suffered from severe deficits in both music perception and production that is generally associated with deficits in language perception and production. This pattern of results is consistent with the idea of a general dysfunction of the auditory-motor loop. An impairment of the auditory-motor loop has already been proposed to account for the musical deficits in congenital amusics (Mandell et al., 2007). The auditory-motor loop involves both the posterior sensory cerebral area and the anterior motor areas connected via the arcuate fasciculus (Hickok and Poeppel, 2004). An abnormal functioning of this fronto-temporal network could be responsible for the musical deficits of amusics (Hyde et al., 2006, 2007; Albouy et al., 2013). More specifically, in a diffusion tensor tractography study, Loui et al. (2009) proposed that tone deafness could be related to an abnormally reduced right arcuate fasciculus connectivity, although this result may be dependent on the fiber tracking algorithm used by the authors (Chen et al., 2015). Similarly, two recent studies also reported abnormal fronto-temporal connectivity in children with SLI (Verhoeven et al., 2012; Roberts et al., 2014). Roberts et al. found a reduced mean diffusivity restricted to the left arcuate fasciculus, whereas Verhoeven et al. found a reduced fractional anisotropy in both the left and right arcuate fasciculus of children with SLI. In the latter study, the reduction in the fractional anisotropy also correlated with the language deficits of the children. Our findings thus seem to be consistent with a bilateral reduction of fronto-temporal connectivity that would cause deficits in the perception and production of both language and music.

All together, the present study is, to our knowledge, the first to report severe singing disabilities in children with SLI. We showed that most of the children with SLI that we tested, in addition to their verbal disabilities, exhibited deficits in the musical domain at both the receptive and expressive levels. These deficits affecting both the music and the language functions are compatible with a more general dysfunction of the auditory-motor loop. Because of the heterogeneity in the population of children with SLI, this interpretation may not account for all cases. In fact, in the present study, two children with SLI showed no deficits in musical abilities. By definition, the diagnosis of SLI is based on the existence of language impairments that cannot be explained by more general cognitive of neurological abnormalities. One can thus argue that, stricto sensu, the children with SLI who display deficits in musical abilities do not fulfill the criteria of “specific” language impairment. Further investigation of musical abilities in larger groups of children with SLI is needed before one can conclude that impaired music abilities should be part of the exclusion criteria for a study on children with SLI.

### Conflict of interest statement

The authors declare that the research was conducted in the absence of any commercial or financial relationships that could be construed as a potential conflict of interest.
